# Neoadjuvant chemotherapy with weekly cisplatin and paclitaxel followed by chemoradiation for locally advanced cervical cancer

**DOI:** 10.1186/s12885-023-10517-x

**Published:** 2023-01-14

**Authors:** Jing Li, Ya Li, Huafeng Wang, Lifei Shen, Qun Wang, Siqi Shao, Yuhong Shen, Haoping Xu, Hua Liu, Rong Cai, Weiwei Feng

**Affiliations:** 1 Department of Obstetrics and Gynecology, Ruijin Hospital, Shanghai Jiaotong University School of Medicine, Shanghai, 200025 People’s Republic of China; 2 Department of Radiology, Ruijin Hospital, Shanghai Jiaotong University School of Medicine, Shanghai, 200025 People’s Republic of China; 3 Department of Pathology, Ruijin Hospital, Shanghai Jiaotong University School of Medicine, Shanghai, 200025 People’s Republic of China; 4 Department of Radiotherapy, Ruijin Hospital, Shanghai Jiaotong University School of Medicine, Shanghai, 200025 People’s Republic of China

**Keywords:** Dose-dense, Neoadjuvant chemotherapy (NACT), Weekly cisplatin/paclitaxel, Concurrent chemoradiation (CCRT), Locally advanced cervical cancer (LACC)

## Abstract

**Background:**

Currently, the standard treatment for locally advanced cervical cancer is concurrent chemoradiation (CCRT). Forty percent of patients present with disease recurrence. This study aims to investigate the feasibility, safety and efficacy of neoadjuvant chemotherapy (NACT) with weekly cisplatin and paclitaxel (TP) followed by CCRT.

**Methods:**

We are conducting a phase III trial comparing the efficacy and side effects of patients with cervical cancer (FIGO 2018 stage IIB to IVA) who were assigned to four cycles of NACT with cisplatin (40 mg/m^2^) and paclitaxel (60 mg/m^2^) weekly followed by CCRT or CCRT alone. In this report, we studied the medium-term effect of 50 patients enrolled in the NACT + CCRT arm. The primary endpoints were the response rate post-NACT and 12 weeks post-CCRT evaluated by MR/CT based on RECIST v 1.1. The secondary endpoints were 3-year OS (overall survival) and PFS (progression-free survival) measured by the Kaplan–Meier method.

**Results:**

Among 50 patients enrolled in the NACT + CCRT arm, the complete and partial response rates were 10.4% and 68.8%, post-NACT. Twelve weeks after treatment completion, the complete response rate was 72.0%, whereas the total response rate (complete and partial response) was 90.0%. After a median follow-up of 28 months, the 3-year OS rate was 83.9%, and the 3-year PFS rate was 73.6%. NACT response was related to superior PFS and OS compared with NACT nonresponse (*P* < 0.01). Late AEs were exiguous, while early AEs mainly included myelosuppression and gastrointestinal AEs.

**Conclusions:**

This study showed a good response rate achieved by dose-dense weekly cisplatin and paclitaxel followed by standard CCRT. The treatment regimen is feasible, as evidenced by the acceptable toxicity of NACT and by the high compliance with radiotherapy.

**Trial registration:**

Protocol version number and date.

Chinese clinical trial registry, ChiCTR1900025327; http://www.chictr.org.cn. Registered 24 August 2019. Retrospectively registered, medresman.org.cn/ChiCTR1900025326.

The date recruitment began 01–01-2019.

## Background

Cervical cancer, a considerable health crisis for women, is the fourth most common cancer worldwide and the fourth leading cause of cancer death [1]. In developing countries, however, it remains the second most common cause for both cancer incidence and mortality [2]. Also, it is the leading cause of cancer-related death in women in eastern, western, middle, and southern Africa [3]. The standard treatment for locally advanced cervical cancer (LACC) is concurrent chemoradiation (CCRT). However, the overall survival (OS) for stage IIB and III-IV cancer is approximately 60–65% and 25%-50%, respectively, which are frustratingly low [4]. Therefore, developing new treatment strategies to improve survival is imperative.

Neoadjuvant chemotherapy (NACT) plays a yet unproven role in cervical cancer treatment, particularly when followed by CCRT, where data are scarce [5]. Traditional triweekly (once every 3 weeks) regimens of NACT followed by CCRT may not be superior to CCRT alone for the treatment of LACC [6–8]. Weekly regimens of NACT followed by CCRT may be superior to CCRT alone [6, 9, 10]. Moreover, particularly in developing countries, the incidence of advanced cervical cancer is high, and access to radiotherapy facilities is limited [11]. These factors result in a delay in treatment initiation, contributing to a worse prognosis [12]. If the benefit of NACT is demonstrated, this may reduce delays in treatment and improve outcomes in communities with scarce resources.

We are conducting a phase III trial comparing the efficacy and side effects of patients with cervical cancer (FIGO 2018 stage IIB to IVA) who were assigned to four cycles of NACT with cisplatin (40 mg/m^2^) and paclitaxel (60 mg/m^2^) weekly followed by CCRT or CCRT alone. In this report, we studied the medium-term effect of 50 patients enrolled in the NACT + CCRT arm. The goal of this interim analysis is to confirm the efficacy and adverse events (AEs) of NACT in cervical cancer patients. If the efficacy is significantly worse than CCRT alone, or the AEs are serious, we should terminate this study.

## Methods

### Patients and study design

A phase III trial is being conducted in Shanghai Jiaotong University Medical School affiliated with Ruijin Hospital [13]. This trial will recruit 300 patients. Patients with IIB-IVA cervical cancer were randomly allocated in a 1:1 ratio to NACT with weekly cisplatin and paclitaxel followed by CCRT or to CCRT alone. The randomization scheme prepared by the study statistician using a random number table, with the results entered by a study manager not involved with patient recruitment. In this study, we studied the medium-term effect of 50 patients enrolled in the NACT + CCRT arm from Jan 2019 to Dec 2021 who were followed up until Mar 2022.

Patients were eligible if they presented with stages IIB to IVA histologically confirmed squamous carcinoma, adenocarcinoma or adeno-squamous carcinoma of the cervix. Tumor staging was assessed according to the 2018 International Federation of Gynecology and Obstetrics staging system. All patients underwent a biopsy, examination and imaging to complete the staging as detailed below. For American Joint Committee on Cancer staging, pelvic magnetic resonance imaging (MRI; or pelvic computed tomography [CT] scan if MRI was contraindicated), abdominal CT scan, and chest radiography were performed. PET/CT (18-fluoro-2-deoxy-D-glucose positron emission tomography [18F FDG-PET] with computed tomography) was performed in the majority of patients. Glomerular filtration rate (GFR), computed tomography urography (CTU), cystoscopy and sigmoidoscopy were performed when necessary. If staging differed between the two staging systems, the higher stage was recorded.

Additional key inclusion criteria were Eastern Cooperative Oncology Group (ECOG) performance status 0 to 2, age 18 to 75 years, and adequate organ function. Measurable disease was evaluated by Response Evaluation Criteria in Solid Tumors (RECIST) version 1.1 [14]. Patients were excluded if they were pregnant or breast feeding, had a previous pelvic malignant disease treated with chemotherapy or radiotherapy, had an acute infection, or had an uncontrolled severe medical condition.

The study was approved by Shanghai Jiaotong University School of Medicine affiliated with the Ruijin Hospital Ethics Committee (N-2018–239). Signed informed consent was obtained from all patients.

### Treatment

NACT consisted of intravenous paclitaxel 60 mg/m^2^ on Days 1, 8, 15 and 22 and cisplatin 40 mg/m^2^ on Days 1, 8, 15 and 22. Within 2 weeks after NACT, patients were treated subsequently by CCRT as soon as hematological recovery permits.

CCRT consisted of weekly cisplatin 40 mg/m^2^ for 5–6 weeks concurrent with radiotherapy. Pelvic external beam radiation therapy was delivered at a dose of 45 Gy in 25 fractions. For all patients who had adequate geometry for intracavitary brachytherapy, it was delivered in 3 weekly fractions (total dose of 24 Gy). Brachytherapy was given following the completion of external beam radiation therapy. Patients received image-guided adaptive brachytherapy (IGBRT) with a high-risk clinical target volume (HRCTV) of 85 Gy in 3–4 fractions. Extended fields were used to treat positive common iliac and/or para-aortic lymph nodes with a total dose of 55–60 Gy. Radiotherapy was planned based on prechemotherapy status, without any dose or field reduction regardless of tumor response.

Treatment was discontinued in cases of disease progression, withdrawal of consent, or unacceptable toxicity. For hematologic toxicity, both drugs were delayed if neutrophils were < 1.5 × 10^9^/L and/or platelets were < 75 × 10^9^/L on the day of treatment and restarted until hematologic improvement to grade 1 was achieved. NACT would be discontinued if myelosuppression could not improve to grade 1 within 7 days. The doses of paclitaxel and cisplatin were reduced by 50% if neutrophils were (0.5–1.0) × 10^9^/L and/or platelets were (25–50) × 10^9^/L. In the event of further hematological toxicity (neutrophils < 0.5 × 10^9^/L and/or platelets < 25 × 10^9^/L), NACT was discontinued. Patients with a significant hypersensitivity reaction to paclitaxel or cisplatin were withdrawn from the study. If NACT is discontinued, patients proceeding to CCRT will commence when hematologic improvement to grade 1 was achieved. In selected patients who presented contraindications to concurrent cisplatin after treatment initiation as a result of renal dysfunction, reduction of the dose was acceptable.

### Outcome assessments

Patients were assessed at baseline, during NACT and CCRT, and during follow-up. A gynecologic examination was performed with an oncologic cytology test and HPV (human papillomavirus) test. Adverse events (AEs) were assessed at each visit per the National Cancer Institute Common Terminology Criteria for Adverse Events (CTCAE version 5.0). Early and late AEs were defined as those that occurred within and after 3 months of treatment completion, respectively.

Full blood counts, tumor markers, and pelvic MRI (or pelvic CT scans if MRI was contraindicated) were performed at baseline, 7–10 days after NACT and 3 months after the end of CCRT, followed by intervals of every 3 months for 2 years and every 6 months thereafter. Further radiological assessments, such as PET-CT and abdominal and chest CT scans, were conducted as clinically indicated. Response and progression were assessed according to RECIST version 1.1.

### Statistical analysis

The primary endpoint of the study was the response rate 12 weeks after completing all treatments. The response rate to NACT was also assessed. Adverse events were based on the maximum toxicity grade for each type of event.

The secondary endpoints were 3-year OS and PFS. OS and PFS were measured from the date of study registration until disease progression or death as a result of any cause. Patients who did not experience disease progression or death were censored at the time of last follow-up.

The Kaplan–Meier method was used to estimate the survival function. Survival curves were compared using the log-rank test. Quantitative variables were compared using *a t* test. Qualitative variables were compared using the *x*^2^ test or Fisher’s exact test. *P* < 0.05 was considered statistically significant. Statistical analyses were performed using GraphPad Prism version 6.02 software (GraphPad Software, La Jolla California USA, www.graphpad.com).

## Results

### Patient population

In total, 50 patients finished the entire therapeutic schedule from Jan 2019 to Dec 2021 and were followed up until Mar 2022. These patients were included in both the efficacy and safety populations. The median age was 53 years (range, 35 to 68 years). Patients presented with stage IIB (28.0%), IIIA (2.0%), IIIB (2.0%), IIIC1 (56.0%) and IIIC2 (12.0%) disease (FIGO 2018). Squamous cell carcinoma was the most common histology (92.0%), whereas adenocarcinoma was seen in 6.0%, and adenosquamous was seen in 2.0%. The median cervical mass size was 5.16 ± 1.17 cm. Most patients underwent PET-CT during staging (92.0%) and assessment (74.0%). Only 2 patients had a contraindication to MRI, and a pelvic CT scan was performed instead.

### Treatment compliance

During NACT, 4 patients conducted 3 cycles chemotherapy and discontinued the 4^th^ cycle neoadjuvant chemotherapy because of grade 4 myelosuppression and could not improve to grade 1 within 7 days. Two patients delayed the beginning of chemoradiation for approximately 3 weeks (one had grade 4 neutropenia with high fever) and 4 weeks (the other had grade 4 hepatic insufficiency).

All patients started radiotherapy. The number of cisplatin weekly doses concurrent with radiotherapy was four to five. Only 7 patients reduced cisplatin to three times (3 had grade 4 neutropenia, 1 had grade 4 thrombocytopenia, 2 had hepatic insufficiency and 1 had renal insufficiency). Hepatitis virus (hepatitis B/C) was checked before treatment and at the time of hepatic insufficiency, and the results were all negative. Two patients discontinued brachytherapy (one discontinued brachytherapy because of unrecovered grade 4 neutropenia that could not be improved, another postponed brachytherapy for 2 weeks because of a large amount of vaginal bleeding with severe anemia and thrombocytopenia).

### Efficacy analysis

The response rate is shown in Table 1. Fifty patients were treated with neoadjuvant chemotherapy, and 48 were assessed. Because of COVID-19 pandemic, 2 patients lost the opportunity for the assessment. Forty-six (92%) patients received 4 cycles of NACT, while four received 3 cycles because of grade 4 myelosuppression and could not be improved to grade 1 within 7 days. In the imaging assessment, 7–10 days after NACT, 79.2% of patients responded; complete response (CR), partial response (PR), and stable disease (SD) were observed in 10.4%, 68.8%, and 20.8% of patients, respectively. Of note, no patient presented with disease progression (PD) at this time point.

**Table 1:** Response to treatment

Response	Post NACT	Post CCRT	Post NACT			Post CCRT		
			FIGO Stage			FIGO Stage		
No. of patients (%)	(*n* = 50)	(*n* = 50)	IIB-IIIB (*n* = 16)	IIIC1 (*n* = 28)	IIIC2 (*n* = 6)	IIB-IIIB (*n* = 16)	IIIC1 (*n* = 28)	IIIC2 (*n* = 6)
CR	5 (10.4)	36 (72.0)	2 (12.5)	2 (7.1)	1 (16.7)	14 (87.5)	18 (64.3)	4 (66.7)
PR	33 (68.8)	9 (18.0)	12 (7.5)	17 (60.7)	4 (66.7)	2 (12.5)	6 (21.4)	1 (16.7)
SD	10 (20.8)	0 (0)	1 (6.3)	10 (35.7)	1 (16.7)	0 (0)	0 (0)	0 (0)
PD	0 (0)	5 (10.0)	0 (0)	0 (0)	0 (0)	0 (0)	4 (14.3)	1 (16.7)
Response rate (CR + PR)	38 (79.2)	45 (90.0)	14 (87.5)	19 (67.9)	5 (83.3)	16 (100)	24 (85.7)	5 (83.3)
Assessment not done	2 (4.0)	0 (0)	1 (6.3)	1 (3.6)	-	-		

Abbreviations: *CR* complete response, *PR* partial response, *SD* stable disease, *PR* progressive disease

All 50 patients received EBRT followed by brachytherapy and were evaluated. Thirty-six (72.0%) patients achieved CR, and 9 (18.0%) achieved PR 3 months after CCRT. The complete responders after NACT continued to be in CR following CCRT. Twenty-six PR and 3 SD at the end of NACT were converted into CR following CCRT. One PR and 4 SD at the end of NACT were converted into PD (5/50, 10%) following CCRT (two patients developed pulmonary and intestinal membrane metastasis, one patient developed intestinal membrane metastasis, one patient developed vaginal metastasis and another developed liver metastasis). Twelve weeks after treatment completion, CR rates were 87.5%, 64.3% and 66.7% in patients with stage IIB-IIIB, IIIC1 and IIIC2 disease, respectively.

### Follow-up

Patients were followed up in an outpatient clinic every 3 months in the first 2 years and then at 6-month intervals. The median follow-up was 28 months (range 6–39 months, 23.90 ± 10.23 months). This time was calculated from the beginning of treatment. Ten patients developed recurrence or metastasis, and 5 patients died from the disease.

Ten cases of recurrence and metastases are presented as follows (several patients had multiple metastases) (Table 2). Five had lymphatic metastases (para-aortic, pelvic, inguinal, left diaphragmatic foot, left collarbone lymphatic metastases), and the others had metastases in the lung, mesentery, pelvis, vagina and liver.

**Table 2:** Initial site of recurrence and outcomes of the patients who presented with disease recurrence during follow-up

No	Age	Histology	FIGO	Response to treatment		Recurrent and metastases		Treatment Compliance	Current situation
	(y)		Stage	Post NACT	Post CCRT	PFS ^a^ (m)	Region	(Reason)	OS ^a^ (m)
1	66	SC	IIIC1r	SD	CR	31	Lymphatic metastases (para-aortic and left diaphragmatic foot)	/	Alive (39)
2	54	SC	IIIC1r	SD	PD	7	Metastases in the lung and mesentery	Reduced cisplatin cycle concurrent with radiotherapy to 3 (hepatic insufficiency)	Dead (12)
3	40	SC	IIIC1r	SD	PR	20	Metastases in the pelvis	/	Dead (31)
4	53	SC	IIB	PR	CR	12	Lymphatic metastases (para-aortic and parailiac vascular)	Reduced NACT cycle to 3 (myelosuppression) Discontinued brachytherapy (grade 4 neutropenia that could not be improved)	Alive (25)
5	35	SC	IIIC1r	SD	PD	6	Lymphatic metastases (left collarbone, para-aortic, parailiac vascular) and liver metastases	/	Dead (10)
6	60	ASC	IIIC2r	CR	CR	13	Local recurrence and lymphatic metastases (para-aortic, pelvic and inguinal)	/	Alive (19)
7	57	SC	IIIC1r	SD	PD	7	Metastases in the lung, mesentery and lymphonodus (inguinal)	Delayed beginning of chemoradiation (grade 4 neutropenia with high fever)	Alive (15)
8	66	SC	IIIC2r	PR	CR	10	Metastases in the vagina	/	Dead (12)
9	43	SC	IIIC2r	SD	PD	6	Metastases in the lung and mesentery	Reduced cisplatin cycle concurrent with radiotherapy to 3 (thrombocytopenia) Postponed brachytherapy for 2 weeks (a large amount of vaginal bleeding with severe anemia and thrombocytopenia)	Dead (14)
10	50	SC	IIIC1r	PR	PD	6	Metastases in the vagina	Delayed beginning of chemoradiation (grade 4 hepatic insufficiency)	Alive (10)

^a^This time was calculated from the beginning of treatment

*SC* squamous carcinoma, *ASC*adenosquamous carcinoma

### PFS and OS

With a median follow-up of 23.8 months, the 3-year PFS rate was 73.6%, and the 3-year OS rate was 83.9%. In all, 5 patients died due to progressive disease at the time of analysis. Among those, four patients were assessed as SD at the end of NACT, while 3 were assessed as PD at the end of CCRT. The five patients developed metastases (metastasis in the liver, pulmonary, intestinal membrane and pelvic) and died at 10–31 months (median survival time 12 months) from the beginning of treatment.

**Fig. 1:**
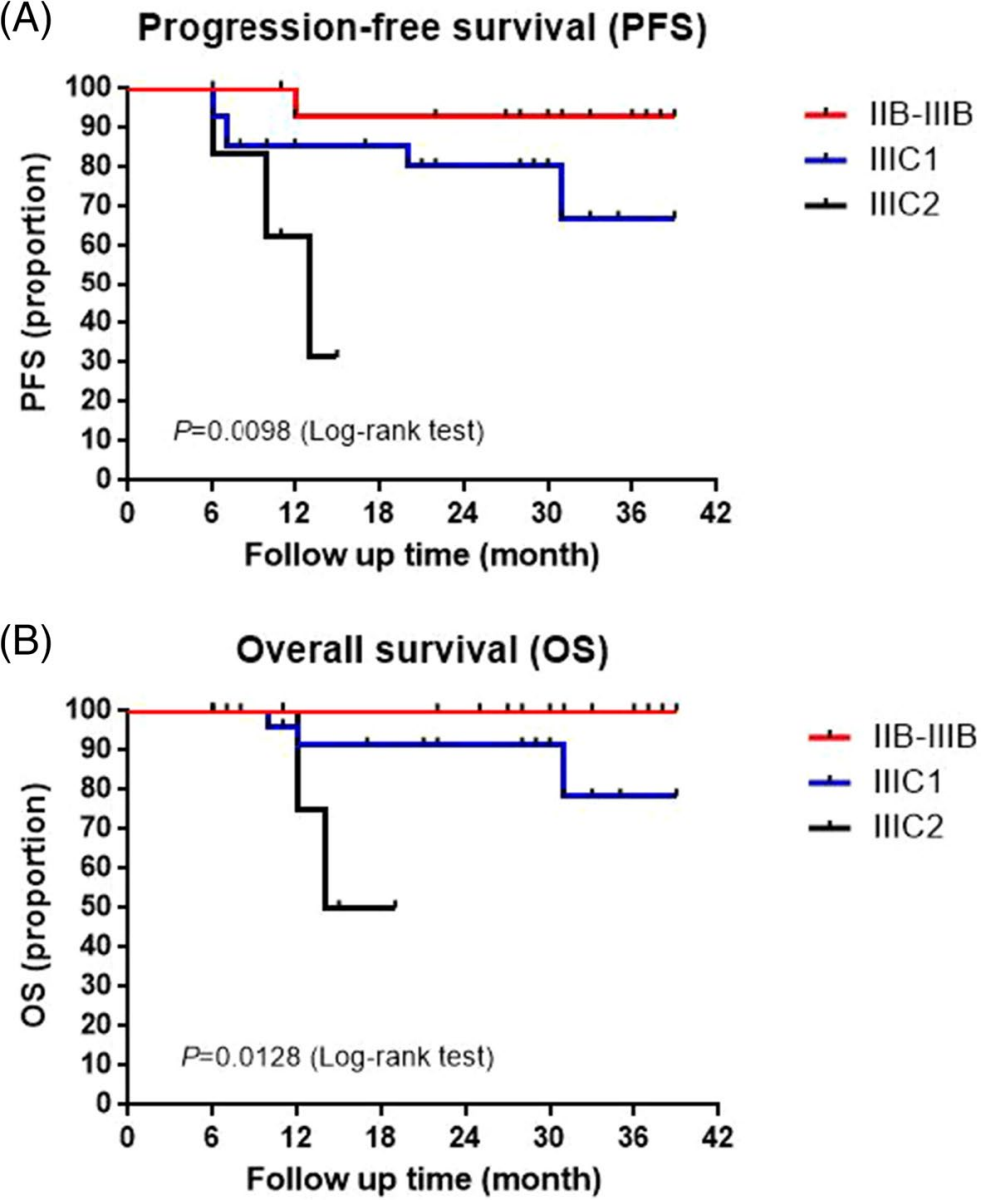
Kaplan–Meier curves for (**A**) progression-free survival (PFS) and (**B**) overall survival (OS). The results presented in the curves refer to the comparison between different FIGO stage survival curves. Patients presented with stage IIB-IIIB (*n* = 16), IIIC1 (*n* = 28) and IIIC2 (*n* = 6) disease

Earlier FIGO stage was related to superior PFS (*P* = 0.0098; Fig. 1A) and OS (*P* = 0.0128; Fig. 1B). The three-year PFS rates of IIB-IIIB, IIIC1 and IIIC2 were 92.9%, 67.0% and 31.2%, respectively. The 3-year OS rates of IIB-IIIB, IIIC1 and IIIC2 were 100%, 78.4% and 50%, respectively. The mPFS (median PFS) and mOS (median OS) of FIGO IIIC2 were 13 months and 23.5 months, respectively. The mPFS and mOS of FIGO IIB-IIIC1 were undefined.

**Fig. 2:**
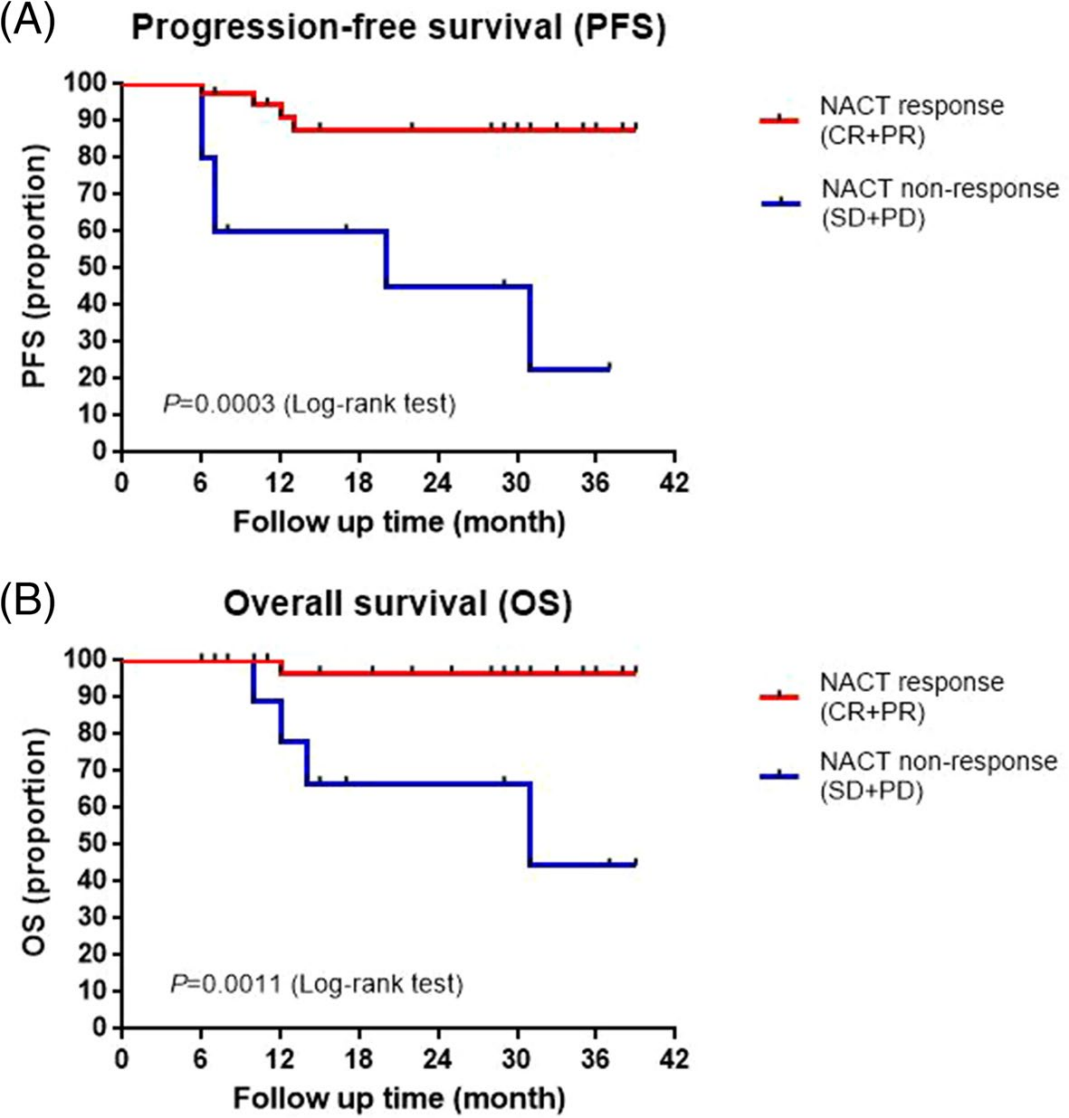
Kaplan–Meier curves for (**A**) progression-free survival (PFS) and (**B**) overall survival (OS). The results presented in the curves refer to the comparison between NACT response (CR + PR) (*n* = 38) and nonresponse (SD + PD) (n = 10) survival curves. NACT, neoadjuvant chemotherapy

NACT response (CR + PR) was related to superior PFS, with 3-year PFS rates of 87.7% vs. 22.5% in NACT nonresponders (SD + PD) (hazard ratio, 0.14; 95% CI, 0.009–0.246; *P* = 0.0003; Fig. 2A). NACT response was also associated with a higher OS (3-year OS rate, 96.7% vs. 44.4%; hazard ratio, 0.07; 95% CI, 0.003–0.237) than NACT nonresponse (*P* = 0.0011; Fig. 2B). The mPFS and mOS of NACT-nonresponsive patients were 20 months and 31 months, respectively, and NACT-responsive patients remained undefined.

### Safety

Adverse events according to NCI CTCAE v5.0 are summarized in Table 3.

**Table 3:** Adverse events, classified according to NCI CTCAE v5.0 the Common Terminology Criteria for Adverse Events (CTCAE) version 5.0, based on the worst grade for each patient and each type of toxicity

No. of patients (%)	During NACT		During CCRT		During follow-up
Toxicity	Any Grade	Grade ≥ 3	Any Grade	Grade ≥ 3	Grade ≥ 3
Any hematological toxicity	41 (82.0)	20 (40.0)	48 (96.0)	26 (52.0)	1 (2.0)
Anemia	18 (36.0)	3 (6.0)	38 (76.0)	19 (38.0)	1 (2.0)
Neutropenia	35 (70.0)	18 (36.0)	42 (84.0)	12 (24.0)	0 (0)
Thrombocytopenia	3 (6.0)	0 (0)	25 (50.0)	8 (16.0)	0 (0)
Any non hematological toxicity	49 (98.0)	16 (32.0)	43 (86.0)	16 (32.0)	1 (2.0)
Nausea	43 (86.0)	4 (8.0)	31 (62.0)	7 (14.0)	0 (0)
Vomiting	30 (60.0)	6 (12.0)	19 (38.0)	1 (2.0)	0 (0)
Constipation	14 (28.0)	1 (2.0)	12 (24.0)	0 (0)	0 (0)
Diarrhea	14 (28.0)	3 (6.0)	19 (38.0)	4 (8.0)	1 (2.0)
Fatigue	36 (72.0)	7 (14.0)	37 (74.0)	10 (20.0)	0 (0)
Alopecia	45 (90.0)	-	36 (72.0)	-	-
Anal pain	0 (0)	0 (0)	18 (36.0)	3 (6.0)	1 (2.0)
Peripheral Neuropathy	3 (6.0)	0 (0)	15 (30.0)	0 (0)	0 (0)
Creatinine Elevation	1 (2.0)	0 (0)	10 (20.0)	0 (0)	0 (0)
Hepatic Enzyme Elevation	1 (2.0)	1 (2.0)	8 (16.0)	2 (4.0)	0 (0)

Adverse event that occurred during treatment and within 14 days of treatment completion

The most common early AEs were myelosuppression, GI symptoms and fatigue.

During NACT — In all, 40% had a grade 3 or 4 hematological toxicity during NACT, and 32% had a grade 3 or 4 nonhematological toxicity. Four patients conducted 3 cycles chemotherapy and discontinued the 4^th^ cycle NACT. Two patients delayed the initiation of chemoradiation therapy for about 3 and 4 weeks.

During CCRT — A total of 52% of patients had grade 3 or 4 hematological toxicity during CCRT. Grade 3 or 4 nonhematological AEs accounted for 32%. Fatigue (14%) and vomiting (12%) were the most common adverse events. Two patients had grade 3/4 liver function impairment during treatment. Seven patients reduced cycles of concurrent cisplatin during CCRT because of grade 4 myelosuppression or grade 3/4 nonhematological toxicity and could not be improved to grade 1 within additional 7 days after the scheduled treatment date. Two patients discontinued brachytherapy.

Late toxicities were mainly low grade and included anemia, diarrhea, and anal pain.

## Discussion

Since 1999, the standard treatment of locally advanced cervical cancer (LACC) has been pelvic radiation with concurrent cisplatin, with an absolute improvement of 12% in overall survival compared with radiotherapy alone [15]. It is important to investigate better treatment strategies considering that approximately 40% of patients experience recurrence within 5 years.

NACT (paclitaxel and carboplatin once every 3 weeks for three cycles) followed by radical surgery resulted in inferior disease free survival compared with cisplatin-based concomitant chemoradiation in locally advanced cervical cancer [16]. NACT before definitive radiotherapy has been generally perceived as not beneficial or even detrimental because of the greater toxicity of the chemotherapy regimen, higher recurrence rate, inferior PFS and lower survival rate [8, 17, 18].

Previous trials investigating the theory of accelerated repopulation suggested that in rapidly proliferating cancer, especially the protracted schedules, there is a large gap between completing chemotherapy and radiotherapy, suboptimal regimens are used, and tumor regrowth may be accelerated after chemotherapy, thereby limiting the effectiveness of NACT [19, 20]. However, data seemed to indicate the importance of dose-intensity and cycle lengths [6]. Cervical tumors are rapidly proliferating with a median doubling time of only 4–4.5 days and a high growth fraction [21, 22]. After a few cell divisions, the tumor volume may be restored, but the tumor cells may be less sensitive to chemotherapy and potentially to conventional radiotherapy due to the changed growth kinetics. With these assumptions, the schedule of chemotherapy may play an important role: a short and high dose-intense chemotherapy cycle seems optimal to minimize tumor repopulation with resistant cells.

Trials using short cycle chemotherapy with a chemotherapy cycle length of < 14 days and cisplatin dose intensities ≥ 25 mg/m^2^ per week appear to be associated with an improvement in survival compared with those using a more prolonged cycle interval [6]. The initial results from two phase II studies [9, 10] have been reported on patients who received NACT using weekly paclitaxel (60–80 mg/m^2^) and carboplatin (AUC = 2) for 6 weeks followed by CCRT. Following NACT, a response rate of 67.8–72.7% was achieved, mostly partial responses. Post-CCRT, the response rate was approximately 90%. Grade 3–4 hematologic toxicity was observed in approximately 20% of patients. A 3-year overall survival rate of 67% was observed in stage IB2-IVA patients. The observations are encouraging. The approach is now being evaluated in a randomized trial (weekly paclitaxel 80 mg/m^2^ and carboplatin AUC 2 for 6 weeks) (NCT01566240: INTERLACE trial) (http://www.clinicaltrials.gov).

For chemotherapy, cisplatin is widely accepted in metastatic or recurrent cervical cancer management and it remains the most active single agent [5]. The triweekly administration of paclitaxel with cisplatin has been considered the most effective regimen for metastatic cervical cancer for a long time [23]. Though this has been challenged by the GOG 240 study which showed bevacizumab plus chemotherapy (cisplatin or topotecan plus paclitaxel) was superior to chemotherapy regimen [24] and another phase III trial showed that pembrolizumab plus chemotherapy with or without bevacizumab was significantly superior to placebo plus chemotherapy with or without bevacizumab [25] at present. In the GOG study published by Moore et al., the combination of cisplatin and paclitaxel was superior to cisplatin alone with respect to objective response rate, progression-free survival and sustained quality of life [26]. Among patients who had not received prior cisplatin, cisplatin-based chemotherapy might be significantly better than carboplatin-based therapy [27, 28]. Increased doses of cisplatin and paclitaxel are associated with survival improvement [29]. Based on these data, we formed our trial to evaluate the addition of NACT (weekly paclitaxel and cisplatin for 4 weeks) to standard CCRT [13].

The results from our trial confirmed that a short course of dose-dense weekly NACT with paclitaxel and cisplatin followed by radical CCRT is feasible with acceptable toxicity, as it did not compromise chemoradiotherapy, with 92% (46/50) of patients initiating and completing the radiation phase within the required time and 86% (43/50) of patients receiving at least 4 cycles of concomitant cisplatin. The observed response rate to this short course of chemotherapy, as assessed radiologically, was associated with an encouraging outcome. The response rates after NACT (79.2%) and after CCRT (90.0%) in our trial were similar to those reported in other studies [9, 10]. The three-year PFS and OS might be higher than those reported in other studies (studies using dose-dense NACT followed by the CCRT strategy are listed in Table 4 [8–10, 30, 31]).

**Table 4:** Characteristics of the studies (NACT followed by CCRT)

Author	Year	Location	Sample size	Average age	FIGO stage	NACT regimen	Response rate to treatment		Follow-up (months)	Survival	
							Post-NACT (%)	Post-CCRT (%)		3-year PFS	3-year OS
Our trial	2022	China	50	53 (35–68)	IIB-IVA	Weekly cisplatin (40 mg/m^2^) and paclitaxel (60 mg/m^2^) for 4 weeks	CR (10.4%) CR + PR (79.2%)	CR (72.0%) CR + PR (90.0%)	28	73.6%	83.9%
Singh RB [9]	2013	India	28	51 (35–67)	IIB-IVA	Weekly paclitaxel (60 mg/m^2^) and carboplatin (AUC = 2) for 6 weeks	CR (7.1%) CR + PR (67.8%)	CR (85.7%) CR + PR (92.8%)	12	-	-
McCormack M [10]	2013	UK	46	43 (23–71)	IB-IVA	Weekly paclitaxel (80 mg/m^2^) and carboplatin (AUC = 2) for 6 weeks	CR (4.5%) CR + PR (72.7%)	CR (67.4%) CR + PR (90.7%)	39.1	68%	67%
da Costa Samantha Cabral S [8]	2019	Brazil	55	48 (22–69)	IIB-IVA	Every 3 weeks Cisplatin (50 mg/m^2^ *d1) and Gemcitabine (1000 mg/m^2^ *d1, d8) for 3 cycles	CR (12.0%) CR + PR (80.0%)	CR (56.3%) CR + PR (92.7%)	31.7	40.9%	60.7%
Gadducci A [31]	2017	Italy	13	55 (35–63)	IIB-IVA	weekly paclitaxel (80 mg/m^2^) and carboplatin (AUC = 2) for 6 weeks	CR (0%) CR + PR (76.9%)	CR (58.3%) CR + PR (91.6%)	12	-	-

Lymph node involvement tended to be correlated with inferior outcome. In the present study, patients with stage IIB-IIIB had a higher probability of achieving CR (87.5%) than those with stage IIIC1 (64.3%) and IIIC2 (66.7%) 12 weeks after treatment completion. Earlier (stage IIB-IIIB) FIGO stage was related to superior PFS (*P* = 0.0098) and OS (*P* = 0.0128). The mPFS and mOS of FIGO IIIC2 were merely 13 months and 23.5 months, respectively. This result suggested that other effective strategies should be investigated for a subset of patients with stage IIIC disease, especially those with IIIC2 disease.

The use of NACT before radiotherapy could potentially eradicate subclinical distant metastasis, reduce the tumor size and correct pelvic anatomy distortion, and ultimately allow better delivery of radiation. Although the long-term survival benefits compared with CCRT alone remain uncertain, our therapeutic strategy incorporating dose-dense TP chemotherapy and CCRT yielded favorable outcomes. After a median follow-up of 28 months, the 3-year OS rate was 83.9%, and the 3-year PFS rate was 73.6%.

The reasons for a possible detrimental effect of neoadjuvant treatment are unclear. In general, NACT was well tolerated, with only 4% (2/50) of patients experiencing any grade 3/4 late adverse events and no treatment-related deaths. A possible explanation for the detrimental results of a particular population might be the toxicity associated with NACT that compromised the ability to deliver concurrent CCRT.

The delay to initiate definitive chemoradiation because of neoadjuvant treatment might be detrimental. Most of the patients in our trial were treated subsequently by CCRT within 2 weeks after NACT. Two patients delayed the initiation of chemoradiation therapy for 3 and 4 weeks. Grade 3/4 hematological toxicity during NACT was 40%, which was similar to the report by Singh et al. (49.7%) but was considerably higher than that reported by McCormack et al. (11%). Most patients recovered soon after medication or blood transfusion, except one patient (stage IIIC1r) who had grade 4 neutropenia with high fever after NACT and delayed the beginning of chemoradiation for approximately 3 weeks. She was assessed for SD after NACT and PD three months after CCRT (the local lesion was in complete remission but developed metastasis in the lung, intestinal membrane and inguinal lymph node). The majority of nonhematological toxicities were gastrointestinal toxicity, fatigue and alopecia (using cold caps may significantly reduce the occurrence of alopecia [32]), which did not delay subsequent CCRT. However, one had grade 4 hepatic insufficiency after 4 cycles of NACT and delayed the initiation of chemoradiation for 4 weeks. She was evaluated as PR after NACT and PD three months after CCRT (local lesion was reduced but developed metastasis in the vagina and inguinal lymph node). The gap between NACT and CCRT was significantly influenced by the development of grade 3/4 neutropenia during NACT. The use of prophylactic G-CSF may help in maintaining dose intensity in future studies using this treatment protocol.

According to the recommendation [32], patients who took more than 8 weeks to complete radiotherapy might also have detrimental outcomes. There were more adverse events during CCRT (52% of patients had grade 3/4 hematological toxicity, and 32% of patients had grade 3/4 nonhematological toxicity). Thus, the incorporation of additional neoadjuvant chemotherapy into the standard treatment regimens is likely to result in increased toxicity. In our trial, one patient who discontinued brachytherapy for grade 4 neutropenia could not have improved and developed metastasis in the para-aortic and pelvic lymph nodes nine months after treatment. Another patient postponed brachytherapy for 2 weeks due to a large amount of vaginal bleeding with severe anemia and thrombocytopenia and developed metastasis in the lung and intestinal membrane three months after CCRT and died thereafter.

Moreover, in nonresponding patients, there might be inherent resistance to both chemotherapy and radiotherapy. Additionally, delayed access to CCRT might be detrimental. The combined analysis showed that a better clinical response and pathologic response to NACT were associated with favorable PFS and OS [33]. Stable disease post-NACT has also been identified by others as a poor prognostic sign [34]. In our trial, 10 patients (20.8%) had stable disease at the end of NACT, 4 of these patients had progressive disease 3 months post-CCRT, and 2 had progressive disease during follow-up, 4 of which subsequently died from their disease. NACT response was related to superior PFS (HR 0.14; *P* = 0.0003) and OS (HR 0.07; *P* = 0.0011) compared with NACT nonresponse.

Finally, tumor cells may have acquired resistance throughout NACT. Some studies have suggested that previous exposure to cisplatin could result in cross-resistant cellular clones and may lead to increased DNA repair and platinum-induced radioresistance [35–37]. Molecular studies of resistance pathways before and after neoadjuvant treatment may help to inform us about this finding.

## Conclusions

In conclusion, the results from this trial suggested that the addition of four cycles of NACT with cisplatin (40 mg/m^2^) and paclitaxel (60 mg/m^2^) weekly followed by CCRT is feasible and showed a preferable response rate. NACT-responsive patients had superior PFS and OS compared with NACT-nonresponsive patients. Compared with stage IIIC patients treated with weekly NACT + CCRT, stage IIB-IIIB patients have superior CR rate, PFS and OS. It is imperative to continue studying resistance pathways and potentially noncross-resistant treatments to improve outcomes, as well as access to the delivery of high-quality radiotherapy in underserved regions of the world. As the randomized multicenter phase III trial (Chinese clinical trial registry, ChiCTR1900025327) is ongoing, it would be interesting to observe whether this treatment strategy leads to a significant improvement in progression-free survival and overall survival compared to standard CCRT.

## Data Availability

All data generated or analysed during this study are included in this published article.

## Abbreviations

CCRT: Concurrent chemoradiation
NACT: Neoadjuvant chemotherapy
TP: Cisplatin/paclitaxel
LACC: Locally advanced cervical cancer
OS: Overall survival
PFS: Progression-free survival
MRI: Magnetic resonance imaging
CT: Computed tomography
PET-CT: 18-Fluoro-2-deoxy-D-glucose positron emission tomography with computed tomography
GFR: Glomerular filtration rate
CTU: Computed tomography urography
ECOG: Eastern Cooperative Oncology Group
RECIST: Response Evaluation Criteria in Solid Tumors
IGBRT: Image-guided adaptive brachytherapy
HRCTV: High-risk clinical target volume
HPV: Human papillomavirus
AEs: Adverse events
CTCAE: Common Terminology Criteria for Adverse Events
AUC: Area under the curve
CR: Complete response
PR: Partial remission
PD: Progression disease
SD: Stable disease
mPFS: Median progression-free survival
mOS: Median overall survival
